# Linking genetic gains to food security outcomes: An assessment of IRRI’S rice breeding efforts in the Philippines and Indonesia

**DOI:** 10.1007/s12571-025-01632-7

**Published:** 2026-02-27

**Authors:** Lawton Lanier Nalley, Jesse Tack, Alvaro Durand-Morat, Valerien Pede, Donald Villanueva

**Affiliations:** 1 Department of Agricultural Economics, University of Arkansas, 217 AGRI Building, Fayetteville, AR 72701 USA; 2 Department of Agricultural Economics, Kansas State University, Manhattan, KS USA; 3 International Rice Research Institute, Los Banos, The Philippines

**Keywords:** Public breeding, Rice, IRRI, Food security

## Abstract

The International Rice Research Institute (IRRI) runs the largest public rice breeding program in the world, playing a crucial role in ensuring global food security. Using a large dataset containing 12,045 yield observations of IRRI elite lines, we estimate the annual genetic gain attributable to IRRI since the release of IR8, the variety that launched the Green Revolution, and the market impacts in Indonesia and the Philippines. Results indicate that since the release of IR8, the genetic gains from IRRI lines have increased by 0.58% annually. The estimated average annual supply increase through producer adoption of IRRI lines in Indonesia and the Philippines was sufficient to feed an additional 25.14 million people’s annual rice consumption. An important aspect of this study’s findings is to highlight the significant role that public breeders like IRRI play in combating global food insecurity, even in the face of diminishing funding.

## Introduction

Rice (Oryza sativa L.) is one of the world’s major staple crops, providing up to 20% of the global dietary energy and feeding more than 3.5 billion people (Wing et al., 2018). With the release of IR8 in 1966 as a driver of the “Green Revolution,” the International Rice Research Institute (IRRI), which is part of the CGIAR, is recognized for having achieved significant impacts on rice productivity throughout the low-income world (Fuglie & Echeverria, 2024). The global rice supply must increase by approximately 30% before 2050 to meet projected demand from a growing population (Ray et al., 2013). Nearly 60 years after the start of the Green Revolution, genetic improvement for rice yield potential is still considered one of the most necessary, but not sufficient, strategies to meet this increased demand while simultaneously addressing the anticipated impacts of climate change on global rice production (Saito et al., 2021).

The research literature suggests that the current rate of genetic improvement in rice has not been on pace to meet the expected increase in demand of 1.5% or above for the growing global population (Khanna et al., 2022). In practice, genetic gains are just one tool to increase global rice supply and should not be viewed as the only vehicle to meet this demand, as the CGIAR and other agricultural science institutes use a multi-pronged approach to addressing food security. Previous studies have estimated that the rate of genetic gain in IRRI’s global rice breeding program is less than 1% a year (Juma et al., 2021; Kumar et al., 2021; Khanna et al., 2022), which is not sufficient to meet future rice demands. Peng et al. (2000) evaluated varieties originating from the IRRI breeding program (between 1966 and 1995) and found an annual gain of 1%. Brennan and Malabayabas (2011) found that the yield gains from IRRI’s contribution to varietal improvement between 1985 and 2009 have ranged from 1.8% to 13% across locations in Vietnam, the Philippines, and Indonesia. Using a dataset with more recent genetic material (1966–2016) from the IRRI breeding program, Venkatanagappa et al. (2021) estimated an annual gain of approximately 0.5%. Using historical data from the IRRI irrigated rice program, Juma et al. (2021) observed a slowdown in the rate of genetic gain, averaging 0.23% annually for 1964–2014, and plateauing during the last ten years. Fischer et al. (2014) analyzed 13 global case studies in rice-growing environments to estimate and compare growth in yield potential attributed to rice breeding programs. They found the average annual growth rate associated with genetic gains to be 0.8%. While there is empirical evidence for rice yield gains overall, the authors concluded that recent progress in genetic gains is “definitely lacking.”

Cobb et al. (2019) highlighted that advances in statistics, quantitative and population genetics, molecular biology, genomics, phenomics, and most recently, machine learning and artificial intelligence offer the potential to transform rice breeding programs toward a data-rich, evidence-based breeding strategy, which could help increase annual genetic improvement moving forward. While plant breeding is going through a paradigm shift from a traditional to a data-intensive strategy, it is important to quantify this transition, as rice breeding is still primarily publicly driven, unlike maize and soybeans. As such, quantifying the benefits of public breeding is imperative to enhance future research and ensure consistent funding.

All breeding programs have multiple objectives, which include yield gains but also incorporate such aspects as agronomic (fertility and pest management), biological (genetics, physiology), and culinary (target markets, quality) goals, which each program may weigh differently. Genetic gain, nevertheless, remains a robust indicator for all three main components of a breeding cycle: crossing, evaluation, and selection (Ceccarelli, 2015; Cobb et al., 2019; Seck et al., 2023; Huehn 2005).

Public rice breeding programs have served as the foundation of genetic improvement efforts across low- and middle-income countries since the advent of the Green Revolution and the commercial release of IR8 (an IRRI-bred variety). Flagship cultivars developed by IRRI, such as IR36 and IR64, cultivated on over 11 million and 10 million hectares globally in any given year, respectively, have played a transformative role in enhancing food security and livelihoods. These varieties exemplify the sustained global impact of public-sector breeding initiatives in delivering high-yielding, resilient rice cultivars that underpin food systems in the low and middle-income world (CGIAR, 2025). However, funding for public breeding is now facing shortfalls that threaten the critical mass of many breeding programs. Burris et al. (2025) found that 51% of surveyed public plant breeders believed budget shortfalls or uncertainty threatened regional and/or global food security. Public breeders often focus on long-term research in which the payoff may require many years of work, often by many individuals across various areas of the public sector. After development and proof of concept by the public sector, the new products are often commercialized by the private sector with little or no return of funding to the public sector.

IRRI’s research is funded by governments, philanthropic foundations, the private sector, international agencies, and through the CGIAR. Between 2009 and 2022, grant money flowing to IRRI peaked at 119 million (2022 USD) in 2012 and was estimated at 56 million USD in 2022, indicating that it has become more difficult for IRRI and likely most CGIAR centers to secure funding for agricultural research in the low/middle-income world (IRRI., 2010–2022). Providing metrics like genetic gain is important to provide benchmarks and goals and better understand the future of global food security. Donors to IRRI and the CGIAR centers are continually searching for enhanced metrics to justify current and future funding (Gates Foundation, 2014). Simply saying that rice breeding has not done enough to keep up with demand fails to recognize the work breeders have done to combat the myriad of issues that make up global food insecurity. Moreover, it fails to acknowledge the significant rice yield gap (the difference between the potential yield and that obtained by producers) that still exists, estimated at 48% for Asia (Yuan et al., 2022) and ignores the role of research and development in other areas such as agronomy and extension. While rice breeding plays a crucial role in improving productivity, it is only one of the many issues that need to be in place for improving yields. Public rice breeding, like the work conducted at IRRI, can help meet the increased global demand for rice, but only if support is maintained and improved, which will likely be more dependent on alternative metrics other than simply quantifying genetic gains.

Here, we expand on the previous research that focuses on yield gains by estimating the impacts these gains have on the general economy. More specifically, this study focuses on the impact of the IRRI breeding program on enhanced rice volume, price, trade, and welfare for the Philippines and Indonesia since the release of IR8 in 1966. These two countries were chosen due to the importance of rice in their respective economies/food security, as well as their reliance on IRRI germplasm, and the rich data (area by variety and producer prices) available for each. In the last five years (2018–22), Indonesia and the Philippines were the 4th and 8th largest global rice producers, accounting for 9.2% of global rice production. During the same period, Indonesia and the Philippines were the 4th and 6th largest global rice consumers, respectively, accounting for 10.1% of global rice demand (USDA FAS, 2024). In 2021, rice accounted for 38% and 42% of the total caloric intake in Indonesia and the Philippines, respectively (FAOSTAT, 2024). IRRI elite lines (pre-commercialized varieties that embody the culmination of genetic improvement efforts within a breeding program) play an important role in food security for both countries, with 25% and 38% of the 2021 total rice area planted to IRRI-developed genetic material in each country, respectively. This study is novel in its dual focus. First, it quantifies the genetic gains achieved through IRRI’s breeding program. Second, it traces the downstream effects of these estimated gains through producer adoption of IRRI-developed lines and their subsequent impacts on prices, welfare, food security, and trade outcomes. This contribution is significant because equivalent levels of genetic improvement can yield markedly different economic and food security impacts across countries, depending on local production systems, consumption patterns, and market structures. By linking genetic advances from breeding programs to their adoption and broader economic consequences, this research provides a more integrated and comprehensive assessment of the true impact of genetic improvement on agricultural productivity, welfare, and food security.

## Methods and data

One of the fundamental transformations at IRRI is that all its early-stage (initial phases of varietal evaluation) and late-stage (trials conducted after promising lines have passed early evaluations) breeding trials are conducted in partnership with national agricultural research and extension systems (NARES) in the respective target population of environments (TPE). TPE is a set of environmental conditions, including climate, soil types, management practices, and biotic and abiotic stresses, in which a new variety or technology is expected to be adopted and perform well. Thus, a given line may be tested in several countries as long as the TPE is consistent. The promising breeding lines from the Rapid Generation Advance, a breeding technique used to accelerate the development of new crop varieties by shortening the time needed to advance from one generation to the next, at IRRI are multiplied and shipped to the corresponding NARES partner in the regions. Under IRRI’s OneRice strategy (the integrated global breeding and research approach led by IRRI and its partners under the CGIAR RICE program), the standard operating procedures for trialing have been outlined. For this specific breeding pipeline, the number of trials was determined to be six, and the experimental designs were fixed and homogenized across all locations.

### Varietal data

**Fig. 1:**
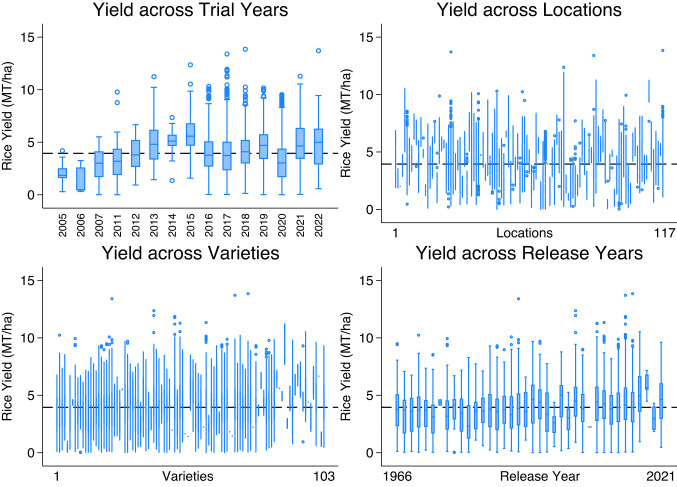
Yield variation across years, locations, varaties and varietal release years

IRRI elite line trials for long-grain, non-fragrant, and non-hybrid rice were collected from 2005 until the 2022 growing season. Rice trial data are combined across 19 countries in the sample (see Fig. 1). Overall, the data has 12,045 unique yield observations spanning 117 locations and 15 years.^1^ The observations are at the variety-location-year level, and we define a field trial as a unique location-year combination. There are 103 varieties in the sample that are included among the 180 field trials. Only lines with two IRRI parents were considered in this study. Thus, our results provide a conservative estimate of actual gains as we fail to capture the benefits of those adopted varieties with 50 percent IRRI genetics (the parent rule) or 25 percent IRRI genetics (the grandparent rule). We also observe the release year of each variety, defined as the year in which the elite germplasm was released by countries for commercial production, and these span from 1966 until 2021 (Fig. 1).

### Econometric modeling

Our statistical model for estimating the yield gains associated with improved genetics leverages field-trial-location (117 locations) and year (15 years) fixed effects. The location-fixed effects to control for all time-invariant factors at the location level (e.g., climate, soil quality), while the year-fixed effects control for pest pressure, weather shocks, and management practices (e.g., increased fertilizer) over time that are common across all locations and varieties. We also consider the inclusion of location-by-year fixed effects as a robustness check, which control for these same features as well as local (trial-level) weather shocks and pest/disease occurrence.

Denoting the log-yield for variety *i* at field-trial location *l* in year *t* by *ly*_*ilt*_, the statistical regression model is given by.

- (1) $${ly}_{ilt}={\alpha }_{l}+{\alpha }_{t}+\beta {rlyr}_{i}+{\varepsilon }_{ilt}$$ 

where the α are the fixed effects, *rlyr*_*i*_ is the release year, and β is the parameter of interest capturing the average yield gain associated with improved genetics. This parameter captures the percentage gain in yields relative to the previous year; for example, an estimate of 0.01 would indicate genetic gains of 1% per year. We cluster the standard errors by year to allow for widespread spatial correlation of the error term within and across all trials.

The release year (*rlyr*) of each variety can be interpreted as the “vintage” of a breeding technology/variety (Arrow, 1962; Nalley et al., 2008; Shew et al., 2018; Traxler et al., 1995). The associated coefficient captures the genetic progression of the IRRI breeding technology across time and is the parameter of focus for measuring the genetic impact of IRRI’s breeding program. A distinction exists between *rlyr*, which varies from 1966 to 2016, and the trial date, which varies from 2005 to 2022. Each variety has a single release year, a year of commercial release, which embodies the breeding technology for that specific year. It is not a time-trend variable as it is time-invariant (i.e., fixed) for each IRRI variety, but rather is modeled similarly to the way that Arrow’s (1962) growth model estimated embodied technology (Traxler et al., 1995). Specifically, Arrow (1962) assigned “serial numbers” of ordinal magnitude to embodied technology in capital. In this study, the variable *rlyr* is the embodied technology for a given release year of the IRRI breeding program. This method is the standard procedure for measuring the impacts of technological change.

### Relative gains

Caution is needed when drawing conclusions about the results for relative genetic gains because the results depend heavily on the varietal baseline used. Using more recent varieties as a baseline results in higher percentage genetic gain values than older varieties, as more recent varieties would typically be associated with higher baseline yields (Ahrends et al., 2018). We use IR8 as the baseline of this study for several reasons. Developed by IRRI in 1966, IR8 was the first semi-dwarf, high-yielding modern rice variety for the tropical irrigated lowlands, making it a natural structural break from varieties before it (Khush et al., 2001). The release of IR8 in 1966 was a structural break in publicly released rice cultivars and, as such, provides an ideal starting point for measuring relative genetic gain.

An important feature of calculating genetic gains associated with a breeding program is to consider the cumulative effects of the program over time since the release of the baseline IR8 variety. That is, the yield gained via the breeding program for a variety released in 2021 is the average yield gain associated with improved genetics (Eq. 1) accumulated from 1966 to 2021 since the release of IR8.

Importantly, this study only estimates the economic impact of genetic gains from the IRRI breeding program via actual varietal adoption. Historical data provided by IRRI was collected from the Philippines and Indonesia from 1991 to 2021 on varietal adoption by area. As such, if a variety released in 1980 were being grown in 2021, we would only associate the cumulative genetic gain from 1966 to 1980. Conversely, if a variety grown in 2021 was released in 2020, we would associate the cumulative genetic gain from 1966 to 2020. In this manner, we weigh the economic impact of a breeding program on both its cumulative genetic gain and its adoption by producers.

### Market impacts

We use a partial equilibrium model of the global rice economy (Durand-Morat & Walies, 2010; Appendix A) to elicit the market impacts (prices, demand, production, and trade) of the gains in rice production in Indonesia and the Philippines attributed to the genetic gains associated with the IRRI breeding program and varietal adoption by producers. The counterfactual question of interest is, “What would the implications be for the global rice market if the gains from IRRI’s rice breeding program in Indonesia and the Philippines had not been realized since the release of IR8?” The scenario is specified by shocking rice yields in Indonesia and the Philippines by the estimated yield change generated by adopting IRRI rice varieties in these countries, and assessing the changes in the market variables of interest. Given the model definition in Appendix A, this is achieved by shocking the output-augmenting technical change variable $AY$ (Eqs. 4 and 5), which implies that the yield gains from IRRI’s rice breeding program have the same efficiency impact across all inputs and factors of production (Hicks-neutral effect). The model calibrates to a database representing the annual average market situation for the period 2013–15 and disaggregates the global rice economy into 76 regional markets and nine rice commodities derived from the combination of rice type (long, medium, and fragrant) and milling degree (paddy, brown, and milled). Therefore, the results should be interpreted as the change in the 2013–15 market situation resulting from the removal of the gains generated by the IRRI breeding program in Indonesia and the Philippines.

## Results

Table 1 shows the RLYR results from all model iterations. A nonlinear specification of RLYR on paddy yield indicated no concavity or convexity in the genetic gains associated with IRRI varieties over time. Thus, genetic gains were assumed to be linear. All five model specifications in Table 1 indicate statistically significant yield gains through time (*P* < 0.01). Regardless of the model specification, the RLYR coefficient is robust and consistent across models. Our guiding principle for selecting a preferred model is to find one that is parsimonious and robust. From a building up perspective, the inclusion of location and year fixed effects are warranted since we know that (i) there are many different agro-climatic features of the trial location that could bias the estimate on release year if not controlled for and (ii) there are evolving changes in management practices and annual biotic and abiotic shocks that could have a similar confounding effect. From there, we considered other alternative models (i.e., AM1-AM4) that either extend it (e.g., location by year FE) or focus on subsamples to investigate how robust the estimate is to other sources of confounding factors.

**Table 1:** Regression results for genetic gain by release year

	Pref Model	AM1	AM2	AM3	AM4
Release year (RLYR)	0.0058***	0.0055***	0.0058***	0.0068**	0.0052***
	−0.0007	−0.0006	−0.0007	−0.0019	−0.0003
Location and Year FE^†^	Y	N	Y	Y	Y
Location-by-Year FE	N	Y	N	N	N
Drop Obs Year < Release	N	N	Y	N	N
Only Philippines Trials	N	N	N	Y	N
Omit Philippines Trials	N	N	N	N	Y
R-sqr	0.368	0.498	0.370	0.327	0.446
*N*	12,045	12,043	11,835	2,956	9,089

^†^Fixed effect

^*^ *p* < 0.05, ** *p* < 0.01, *** *p* < 0.001

The estimate from the preferred model indicates that average yields associated with the IRRI irrigated elite line breeding program increased by 0.58% per year. This result is within the range of previous studies conducted on global rice breeding programs of 0.23% to 1.0% (Juma et al., 2021; Khanna et al., 2022; Peng et al., 2000; Venkatanagappa et al., 2021; and Fischer et al., 2014). We also considered alternative models that allowed the RLYR coefficient to vary across countries. The first one included interactions between country-level dummy variables and RLYR, the results of which suggested the estimate only ranges between 0.51% to 0.58%. The other approach leveraged a multilevel mixed model that included a fixed component for RLYR along with a random slope component by country, which provided a fixed estimate of 0.56% and the random slope had a variance of 4.06 × 10^–9^.

Using the average yield of IR8 in the entire sample of 3.53 t/ha as our base comparison, a 0.58% increase equates to 0.02 t/ha/yr.^2^ While 0.58% annual gain is less than the estimated 1.5% gain needed to feed the rice demand in 2050 (Khanna et al., 2022), it is impressive that since the release of IR8, known as the “miracle rice,” yield gains have not plateaued at the IRRI breeding program and that genetic gains have continued to increase over 55 years.

### Increased supply

Using the RLYR coefficient from Table 1, yearly production gains due to IRRI’s genetic gains were estimated based on the actual hectares planted to IRRI elite varieties for the Philippines (Table 2) and Indonesia (Table 3).^3^ An average of 2.7 million hectares of IRRI elite lines in the Philippines were sown annually between 1991 and 2021. Using the cumulative genetic gain rule combined with the replacement rule (cumulative genetic gains are the difference between an adopted varieties RLYR and 1966 (commercial release of IR8), not the difference between a growing year and 1966), results suggest that on average, the adoption of IRRI elite lines in the Philippines has led to an additional 1.665 million tons of rice annually from 1991 to 2021. From a static point of view (without market reaction to the gain in production) and using annual Filipino producer rice prices (FAOSTAT, 2024), these additional yield gains generate an additional $660.73 million (2022 USD) in average annual revenue for rice producers. From a food security standpoint, assuming the 2021 per capita rice consumption in the Philippines of 211.11 kg in paddy yield equivalent (USDA FAS, 2021), the additional rice rations (additional rice supply divided by yearly per capita rice consumption) associated with the adoption of IRRI elite lines is enough to feed an average of an additional 7.89 million Filipinos, or 6.93% of the population of the Philippines in 2021.

**Table 2:** Additional rice produced, revenue gains (2022 USD/year), and rice rations attributed to the adoption of IRRI elite lines in the Philippines: 1991–2021

Year	Area (ha) to IRRI elite lines^a^	Rice price (2022 USD/t)^b^	Additional rice (t)^c^	Additional revenue (2022 USD)^d^	Additional rations of rice^e^
2021	1,815,928	367.53	1,606,146	590,306,686	7,608,098
2020	1,452,242	381.86	1,212,755	463,102,680	5,744,660
2019	2,292,063	374.55	1,902,647	712,636,396	9,012,585
2018	2,387,722	443.92	1,947,567	864,564,013	9,225,367
2017	2,201,402	431.37	1,782,310	768,835,048	8,442,565
2016	2,383,266	447.51	1,865,927	835,020,870	8,838,647
2015	2,059,915	470.19	1,589,403	747,321,475	7,528,791
2014	2,360,831	558.89	1,793,693	1,002,476,919	8,496,484
2013	2,666,354	501.12	2,004,598	1,004,544,038	9,495,513
2012	2,795,745	489.60	2,088,809	1,022,681,086	9,894,412
2011	2,859,445	455.62	2,065,021	940,864,772	9,781,729
2010	2,407,416	442.36	1,665,536	736,766,463	7,889,422
2009	2,197,717	418.51	1,433,426	599,902,935	6,789,946
2008	2,231,326	433.34	1,397,039	605,392,873	6,617,588
2007	2,534,250	343.13	1,580,400	542,282,733	7,486,146
2006	3,065,868	295.85	1,924,324	569,311,112	9,115,265
2005	3,334,082	283.66	2,091,887	593,384,808	9,908,993
2004	3,540,249	261.21	2,229,076	582,256,874	10,558,835
2003	3,239,993	259.41	1,924,192	499,154,650	9,114,642
2002	3,371,392	278.01	2,024,827	562,922,177	9,591,337
2001	3,310,895	264.88	1,889,673	500,536,623	8,951,130
2000	3,160,205	323.76	1,732,658	560,965,481	8,207,372
1999	3,534,258	353.61	1,879,501	664,610,514	8,902,949
1998	2,555,371	364.47	1,305,432	475,790,919	6,183,659
1997	2,868,255	488.12	1,413,832	690,119,537	6,697,133
1996	3,207,137	578.41	1,502,499	869,060,294	7,117,137
1995	2,569,065	540.76	1,099,026	594,309,465	5,205,941
1994	2,734,996	440.96	1,123,758	495,532,260	5,323,092
1993	2,810,348	403.24	1,190,759	480,161,760	5,640,468
1992	2,766,331	394.03	1,125,681	443,551,952	5,332,200
1991	3,152,333	373.02	1,244,584	464,254,619	5,895,428
Average			1,665,709	660,729,743	7,890,243
Total			51,636,985	20,482,622,033	244,597,534

^a^ IRRI 2024

^b^ Filipinio producer rice price. FAOSTAT 2024

^c^The product of the RLYR coefficient times (RLYR for IRRI variety – RLYR IR8) multiplied by area planted with IRRI variety in the Philippines

^d^The product of additional tons and annual rice price

^e^ Assumning the 192.52 kg (paddy yield equivalent) annual (2021) per capita consumption of rice (Statista, 2024)

**Table 3:** Additional rice produced, revenue gains (2022 USD/year) and rice rations attributed to the adoption of IRRI elite lines in Indonesia: 2002–2021

Year	Area (ha) to IRRI elite lines	Rice price (2022 USD/t)	Additional rice (t)	Additional revenue (2022 USD)	Additional rice rations
2021	2,613,362	353.80	1,630,808	576,979,929	8,470,851
2020	4,031,647	399.74	2,575,690	1,029,606,157	13,378,816
2019	3,500,211	393.22	2,262,493	889,657,305	11,751,987
2018	4,958,504	393.76	3,103,289	1,221,951,220	16,119,309
2017	4,483,170	425.38	2,777,785	1,181,614,217	14,428,553
2016	3,764,060	793.92	2,408,264	1,911,969,218	12,509,164
2015	5,363,080	787.36	3,248,648	2,557,855,538	16,874,341
2014	5,420,731	801.00	3,493,717	2,798,467,499	18,147,295
2013	5,443,308	859.82	3,490,753	3,001,419,578	18,131,900
2012	5,805,932	1,033.63	3,702,016	3,826,514,875	19,229,254
2011	5,757,747	1,055.99	3,660,919	3,865,893,685	19,015,785
2010	5,860,750	371.93	3,660,316	1,361,381,421	19,012,655
2009	6,442,800	262.33	4,025,018	1,055,882,925	20,907,011
2008	7,056,912	352.39	4,494,296	1,583,745,080	23,344,568
2007	7,003,742	362.53	4,228,438	1,532,935,685	21,963,631
2006	6,749,859	311.43	4,228,438	1,316,862,495	21,963,631
2005	6,779,215	291.50	3,786,190	1,103,674,511	19,666,478
2004	6,984,610	252.32	3,740,418	943,782,385	19,428,727
2003	6,149,577	206.91	3,114,324	644,384,869	16,176,628
2002	5,769,503	201.68	2,790,960	562,880,890	14,496,989
Average			3,321,139	1,648,372,974	17,250,879
Total			66,422,783	32,967,459,480	345,017,572

^a^ IRRI 2024

^b^ Indonesian producer rice price. FAOSTAT 2024

^c^The product of the RLYR coefficient times (RLYR for IRRI variety – RLYR IR8) times area planted with IRRI variety in Indonesia

^d^The product of additional tons and annual rice price

^e^Assumning the 192.52 kg (paddy yield equivalent) annual (2021) per capita consumption of rice (Statista, 2024)

A similar story unfolds in Indonesia (Table 3), where there was an average of 5.5 million hectares of IRRI elite lines from 2002 to 2021. Using the same cumulative genetic gain rules applied to the Philippines scenario, this would equate to an average of 3.21 million additional tons of rice annually through the adoption of IRRI lines. Using Indonesian producer prices (FAOSTAT, 2024) and the additional yield via IRRI line adoption, this would lead to an average of $1.65 billion a year (2022 USD) in additional revenue (Table 3). Several things drive the revenue differences between Indonesia and the Philippines, the obvious being area sown to IRRI elite lines, the others are rice price differences received by producers (where the average price in Indonesia was 24.85% higher), and the quickness with which rice producers in each country adopt newer IRRI elite lines. The average age of IRRI adopted lines in 2021 in the Philippines was 13.6 years old, and in Indonesia, it was 24.41 years old,^4^ which affects how the cumulative genetic gain was calculated given that newer varieties have larger cumulative gains. Assuming the 2021 per capita rice consumption in Indonesia of 192.52 kg/yr of paddy yield equivalent (Statasia, n.d.), the additional gains associated with the adoption of IRRI elite lines in Indonesia would provide 17.25 million additional annual rations, the equivalent of 6.30% of the 2021 Indonesian population.

Another way of interpreting these results is the counterfactual case. That is, what would have happened if the IRRI elite line breeding program had ended with the release of IR8? The implicit counterfactual is that rice producers throughout the Philippines and Indonesia would have continued to grow varieties of the vintage and yield of IR8, forfeiting the benefits from more recent releases. However, it is more likely that rice producers would have adopted varieties developed by other breeding programs (national agricultural research and extension systems [NARES], private breeders, etc.). As such, the estimates derived above would overestimate the total benefits of the IRRI rice breeding program, as producers would backfill the loss of the IRRI breeding program with alternative cultivars. Conversely, this study underestimates the holistic benefits of the IRRI breeding program in that it does not quantify the parent and grandparent rule impacts. Without the IRRI breeding program, NARES would likely have less germplasm to make local crosses for release. Further, this study does not estimate the benefits created in terms of pathogen/disease/pest resistance, which has been done in other studies (Nalley et al., 2016; Marasas et al., 2003).

Economists and donors tend to undervalue the opportunity cost of “maintenance breeding” (Marasas et al., 2003). Sparger et al. (2013) found that approximately 40% of agricultural research in the United States is devoted to maintenance. Increasing rice productivity requires increasing effort to “maintain” previous gains. The continually evolving pest and disease complex is one of the main drivers of new varietal releases, and finding new solutions to these issues has been a major objective of IRRI breeding research. Without constantly upgrading resistance by sustained investment in maintenance research at IRRI and other breeding programs, rice yield and stability would likely decline. The substantial economic benefit that accrues from avoided yield losses through maintenance breeding is typically not valued in rice breeding because rice producers do not experience the prevented yield losses, but breeding programs still incur the costs to prevent them. Further, IRRI breeders have continuously worked on improving rice quality (increased milling rates and reduced chalk percentage, specific amylose specifications) from their releases which do not change the paddy yield of rice but can impact the economic return to a producer. Previous studies have shown that marginal improvements in milling genetics have the potential to significantly increase producer revenue without increasing input use or productivity (Nalley & Durand-Morat, 2023). While we do not explicitly estimate maintenance benefits or the enhancement of quality attributes here, we implicitly acknowledge IRRI’s important contribution through its maintenance breeding and quality improvements.

### Market impacts

Table 4 shows the market-wide impact of the benefits generated by the IRRI rice breeding program in Indonesia and the Philippines. The benchmark represents the market condition in 2013–15, and the scenario represents the market situation in 2013–15 without the yield gains generated by the IRRI’s rice breeding program. The yield shocks are estimated considering the additional rice produced because of IRRI’s breeding program in 2021 (Tables 2 and 3) and the total volume of production as reported by FAOSTAT in 2021 (54.4 and 20.0 million metric tons (mmt) in Indonesia and the Philippines, respectively), which translates into a 3.0% and 8.1% decrease in average rice yields in the Philippines and Indonesia, respectively. The nominal and percentage changes reported in Table 4 are estimated relative to the scenario ([benchmark-scenario]/scenario) to frame the discussion in terms of benefits generated by, rather than losses due to the absence of, the IRRI’s rice breeding program.

**Table 4:** Changes in the rice market with the removal of the genetic gains associated with the IRRI rice breeding program in Indonesia and the Philippines

Market variables	Benchmark	Scenario	Change	% change
Indonesia				
Production (mmt, milled basis)	36.20	35.64	0.56	1.6%
Imports (mmt, milled basis)	0.82	1.15	−0.32	−28.0%
Demand (mmt, milled basis)	38.00	37.77	0.24	0.6%
Paddy price at farm gate (US$/mt milled basis)	926	1,038	−112	−10.8%
Milled rice retail price (US$/mt milled basis)	756	842	−86	−10.2%
Value production ($ million)	21,290	23,490	−2,200	−9.4%
Value Consumption ($ million)	27,015	29,909	−2,894	−9.7%
Rice area (1,000 ha)	12,010.0	12,190.8	−181	−1.5%
Philippines				
Production (mmt, milled basis)	11.56	10.88	0.69	6.3%
Imports (mmt, milled basis)	1.48	1.83	−0.35	−19.1%
Demand (mmt, milled basis)	12.90	12.56	0.34	2.7%
Paddy price at farm gate (US$/mt milled basis)	426	482	−56	−11.6%
Milled rice retail price (US$/mt milled basis)	888	988	−100	−10.1%
Value production ($ million)	7,816	8,319	−503	−6.0%
Value Consumption ($ million)	11,462	12,417	−955	−7.7%
Rice area (1,000 ha)	4676.0	4782.4	−106	−2.2%
World				
Import price (US$/mt milled basis)	816	818	−1.80	−0.22%
Global trade (mmt, milled basis)	40.97	41.47	−0.50	−1.21%
Global trade excl. Indonesia and the Philippines	38.66	38.50	0.17	0.44%
Global consumption (mmt, milled basis)	485.76	484.94	0.83	0.17%
Global consumption excl. Indonesia and the Philippines	434.85	434.60	0.25	0.06%

Overall, the results show that adopting IRRI rice varieties in Indonesia and the Philippines resulted in larger production and consumption, lower imports, and lower producer and consumer prices, making rice more affordable for consumers. In Indonesia, adopting IRRI varieties resulted in a 0.56 mmt or 1.6% increase in production and a 1.5% decrease in planted area, a 0.24 mmt or 0.6% increase in consumption, and a 0.32 mmt or 28% decrease in imports. The producer and consumer prices were 10.8% and 10.2% lower, attributable to adopting IRRI varieties. Due to the inelastic nature of rice production and demand, the larger production afforded by adopting IRRI varieties resulted in 9.4% and 9.7% lower values of rice production (revenue) and consumption. It is important to notice that the decrease in production revenue estimated here is dynamic in the sense that market prices are allowed to adjust to the increase in production due to IRRI yield gains, which results in lower unitary production costs. It is also important to highlight that lower production revenues do not mean producer loses. By the zero-profit condition held by the model, producer welfare remains unchanged. Like Indonesia, IRRI’s yield gain benefits in the Philippines resulted in 0.69 mmt or 6.3% higher production and an 11.6% decrease in producer prices. The lower producer prices translated into 10.1% lower consumer prices, which expanded demand by 0.34 mmt or 2.7%. The increased competitiveness (lower prices) of the rice sector created by adopting IRRI’s rice varieties resulted in import substitution amounting to 0.35 mmt, equivalent to a 19.1% decrease in imports. Adopting IRRI varieties made it possible to expand rice consumption and reduce the total cost of rice consumption by 7.7%

At the global level, adopting IRRI rice varieties in Indonesia and the Philippines resulted in a marginal (0.22%) decrease in the average import price and reduced global rice trade by 0.50 mmt or 1.21%. However, trade among all other countries except Indonesia and the Philippines increased slightly. Finally, global rice consumption increased by 0.83 mmt or 0.17%, which amounts to 25.14 million rice rations a year, considering the average per-capita consumption of 67.5 kg/year.

## Conclusions

This study presents the first results investigating the spatial and temporal dynamics of the IRRI rice breeding program in terms of both producer and market impacts through the adoption of IRRI varieties. Our results indicate that since the release of IR8 in 1966, the genetic gains from IRRI lines averaged 0.58% annually. The literature would suggest that this is well below the estimated yield gains (1.5%) needed to feed the global rice demand in 2050. It’s a fallacy to believe that plant breeding is the only component of increasing farm yields. While plant breeding is the backbone of genetic increases, it is part of a myriad of components that result in yield increases. The 1.5% yield gain target should not be shouldered by plant breeders alone but rather through collaboration with all agricultural scientists. For example, agronomists are needed to promote best management practices, agricultural engineers are needed to improve irrigation systems, and agricultural economists are needed to study input markets to provide policy recommendations to ensure affordable access to inputs. Moreover, agricultural extension services must be properly funded to ensure scientific advances reach farmers and bring about fundamental changes. While there is always room for improvement, these genetic gains should be heralded since, if maintained in the future, they will contribute 39% (0.58%/1.5%) of the productivity gain needed to feed the demand for rice of the global population by 2050. As impressive is the fact that these gains have not plateaued, given an environment where public funding becomes increasingly scarce and more competitive.

The yield potential of rice has experienced two significant growth periods that coincide with the introduction of semi-dwarfism and the utilization of heterosis (Zhu et al., 2016). In this study, we do not estimate the gains from the IRRI hybrid breeding program, which warrants future research as the genetic gains from the hybridization of rice has greater potential for yield increases than purebred lines.

Our genetic gain estimates do not capture the gains that countless national agricultural research and extension systems have captured through the increased germplasm pool via IRRI varieties. This is relevant given that IRRI ceased directly releasing varieties for farmers in 1975, and increased testing, evaluation, and release activities have been transferred to the National Agricultural Research and Extension Systems (NARES) rather than being undertaken by IRRI. This reflects a maturing of the roles of IRRI and NARES and is a sign that NARES in these countries have developed into productive and effective organizations in collaboration with IRRI (Brennan & Malabayabas, 2011).

While IR8 is known as the “miracle rice” was part of the start of the Green Revolution, our study focused on what happened to rice yields after its release. In his 1970 Nobel lecture, Norman Borlaug ironically said, “There are no miracles in agricultural production. Nor is there such a thing as a miracle variety of wheat, rice, or maize which can serve as an elixir to cure all ills of a stagnant, traditional agriculture” (Borlaug, 1970), which is why the progression of all breeding programs needs to be consistently evaluated to assess genetic gains and holistic impacts. IRRI breeds rice for countless low and middle-income countries, of which we just focused on two, the Philippines and Indonesia. In 2021, the adoption of IRRI-developed rice varieties generated an estimated additional economic surplus of USD 590.3 million in the Philippines and USD 577.0 million in Indonesia (2022 prices). These benefits are substantial when compared to IRRI’s largest recorded inflow of grant funding, USD 119 million (2022 prices) in 2012, which encompassed all institutional research activities, not solely breeding programs. While these figures underscore the significant economic returns associated with IRRI’s varietal innovations, a comprehensive cost–benefit assessment remains constrained by the unavailability of detailed expenditure data from both IRRI and the collaborating National Agricultural Research and Extension Systems (NARES).

In 2021, IRRI lines and the estimated genetic gains associated with them, and producer adoption accounted for a combined 16.08 million additional rice rations in those two countries alone. Stated another way, the supply increase through producer adoption of and the genetic gains associated with the IRRI breeding program was sufficient to feed an additional 16.08 million people’s per capita rice consumption for a year. The market impacts are equally impressive. We estimated that the genetic gains associated with IRRIs’ breeding program and the ultimate adoption of those lines by Indonesian and Filipino rice producers lowered consumer rice prices by 10.2% and 10.1%, respectively. Notably, the genetic gains estimated in this study can be applied to any other country where IRRI varieties have been used, conditional on having harvested area and production by rice variety. Moreover, these findings can be useful to benchmark the contributions of other rice breeding programs against the largest rice breeding program in the world, which could lead to better program evaluation and, ultimately, help allocate public resources better in the context of diminishing public funding (Burris et al., 2025).

In many crops, such as maize and soya, the private industry has taken over what was recently dominated by public breeding. Four significant drivers have been identified in this ongoing paradigm shift, which has resulted in plant breeding evolving from a traditionally publicly dominated sector to one that now heavily relies on the private sector (Morris et al., 2006). The identified drivers were the commercialization of agriculture, strengthening intellectual property rights, consolidation of the plant breeding industry, and the decline in public development assistance (regional, national, and international). This shift does not assert a misallocation of funds, as the private industry often can innovate quicker, think more entrepreneurially, and respond to changing market conditions faster. This paradigm shift in which human and financial capital now predominately flow into the private sector does beg the question: who will be left to conduct research for the public good where financial gains are difficult to capture? In most crops and most regions of the low and middle-income world, private-sector agricultural research is not likely to generate large impacts on production or social welfare (Evenson & Gollin, 2003). This is why metrics beyond percentage yield increase are important for public breeding institutions. Private companies could have superior genetics and higher yield gains, but if they price themselves outside the reach of poor producers, those gains will never be realized by those who need them the most. A better metric of success should be the combination of genetic enhancements and overall adoption rates of material. This study has shown that the IRRI breeding program has demonstrated that it has increased genetic gain in the face of diminishing funding and held a substantial market share of varietal adoption, 25% and 38% of the 2021 area in Indonesia and the Philippines, respectively.

Since the release of IR8, the global agricultural research landscape has evolved. National agricultural research programs are much stronger, and the private sector is active in rice improvement as well (though mostly in hybrid rice). These public and private programs are developing new rice varieties that often compete directly with IRRI’s varieties. IRRI’s role in rice research continues to evolve as national research programs’ demands change. Two paths seem to be options moving forward. First, IRRI could attempt to maintain a comparative advantage in providing finished varieties because of economies of scale in research (Maredia & Byerlee, 2000) or IRRI could provide advanced breeding material and platform technologies for use by national and private-sector researchers to enhance their productivity. Regardless of the path chosen, an important aspect of this study’s findings is to highlight the significant role that public breeders like IRRI play in global food security. As the private industry plays a more significant role in traditionally dominated public breeding spaces like rice, a paradigm shift is unfolding, shifting from breeding for global food security to breeding for profit. While food security and profitability are not mutually exclusive, the marketability of some breeding traits may service producers in high-income countries more than producers in low-income countries. This study has shown the significant and continuous impact IRRI has had on two countries that rely on public breeding. While the genesis of the Green Revolution started with the release of IR8 over 58 years ago, its genetic footprint is still continuing to battle global food insecurity today through the dissemination of IRRI genetic material across the globe.

## Data Availability

Data is available from authors upon request.

## Appendix 1. RiceFlow model description

The RiceFlow model is a partial, spatial equilibrium model of the global rice economy. It simulates the behavior of the entire rice supply chain, from input markets all the way up to the aggregate final demand, in multiple countries/regions (set $R$) around the world. The production of endogenous rice commodities (set *CE*^5^) is specified as a weak-separable, constant return to scale production function.

1 $${Y}_{c,r}={H}_{c,r}\left\{{G}_{c,r}\left({FAC}_{c,r}\right),{INT}_{c,r}\right\} \forall c\in CE, r\in R$$

where $Y$ represents output, $H$ and $G$ are technology functional forms *FAC*^6^, is the set of factors of production, and INT^7^ is the set of intermediate inputs.

Defining $G$ in $(1)$ as a constant elasticity of substitution (CES) function, the derived demand for factor of production, $QFC$, is

2 $$\begin{aligned}&{QFC}_{f,c,r}\ast{AFC}_{f,c,r}\\&={QVA}_{c,r}\ast{SVA}_{f,c,r}\ast\left[\frac{{PFC}_{f,c,r}}{{PVA}_{c,r}\ast{AFC}_{f,c,r}}\right]^{{-\sigma VA}_{c,r}}\\&\forall f \in FAC, c \in CE,r \in R\end{aligned}$$

3 $${PVA}_{c,r}={\left[\sum_{f}{SVA}_{f,c,r}*{\left(\frac{{PFC}_{f,c,r}}{{AFC}_{f,c,r}}\right)}^{1{-\sigma VA}_{c,r}}\right]}^{\frac{1}{1{-\sigma VA}_{c,r}}} \forall c\in CE, r\in R$$

where $AFC$, $PFC$, and $SVA$ are a factor-, sector-, and region-specific augmenting technical change variable, factor price variable, and cost share in value added, respectively, and $QVA$ and $PVA$ are a sector- and region-specific derived demand and price for the value added composite, respectively. Finally, $\sigma VA$ is the sector- and region-specific elasticity of substitution in value added.

Defining $H$ in $(1)$ as a constant elasticity of substitution (CES) function, the derived demands for intermediate inputs $QIC$, and for the composite value added ${QVA}_{c,r}$, are

4 $$\begin{aligned}&{QIC}_{i,c,r}\ast{AIC}_{i,c,r}\\&=\frac{Y_{c,r}}{{AY}_{c,r}}\ast{SITC}_{i,c,r}\ast\left[\frac{{PIC}_{i,c,r}}{{PY}_{c,r}}\ast{AIC}_{f,c,r}\ast{AY}_{c,r}\right]^{{-\sigma Y}_{c,r}},\\&\forall i \in INT,c \in CE,r \in R\end{aligned}$$

5 $$\begin{aligned}&{QVA}_{c,r}\ast{AVA}_{c,r}\\&=\frac{Y_{c,r}}{{AY}_{c,r}}\ast{SVATC}_{c,r}\ast\left[\frac{{PVA}_{c,r}}{{PY}_{c,r}}\ast{AVA}_{c,r}\ast{AY}_{c,r}\right]^{{-\sigma Y}_{c,r}},\\&\forall c \in CE,r \in R\end{aligned}$$

where $AIC$, $PIC$, and $SITC$ are input-, sector-, and region-specific input augmenting technical change variable, input price variable, and input cost share in total cost, respectively. Furthermore, $AVA$, $AY$, and $PY$, and $SVATC$ are sector- and region-specific value-added augmenting technical change variable, output augmenting technical change variable, output price variable, and value-added cost share in total cost, respectively. Finally, $\sigma Y$ is the sector- and region-specific elasticity of substitution in final output.

The model assumes zero profits in production (Eq. 6) and equilibrium in output markets (Eq. 7i for paddy rice commodities,^8^ and 7ii for other rice commodities^9^).

6 $$\begin{aligned}&{PY}_{c,r}\\&=\frac{{\begin{array}{c}\bigg[{SVATC}_{c,r}*{\left(\frac{{PVA}_{c,r}}{{AVA}_{c,r}}\right)}^{1{-\sigma Y}_{c,r}}\\+\sum_{i}{SITC}_{i,c,r}*{\left(\frac{{PIC}_{i,c,r}}{{AIC}_{i,c,r}}\right)}^{1{-\sigma Y}_{c,r}}\bigg]\end{array}}^{\frac{1}{1{-\sigma Y}_{c,r}}}}{{AY}_{c,r}},\\& \forall c\in CE, r\in R\end{aligned}$$

7i $${Y}_{c,r}={QD}_{c,r}+{\sum }_{s}{QBX}_{c,r,s}+{QK}_{c,r} , \forall c\in CP, r\in R$$

7ii $${Y}_{c,r}={QD}_{c,r}+{\sum }_{s}{QBX}_{c,r,s}, \forall c\in CCP, r\in R$$

where $QD$ represent the volume of output $c$ sold in the domestic market, $QK$ is the change in stocks^10^ of good c, and $QBX$ is the volume of bilateral exports of $c$ from region $r$ to region $s$.

Import demand follows the Armington approach (Armington, 1969), by which imports by source and domestic production are treated as heterogeneous products. Agents first decide on the sourcing of imports (Eq. 8) based on the relative level of prices from each source (Eq. 9).

8 $${QBX}_{c,s,r}={QM}_{c,r}*{SMS}_{c,s,r}*{\left[\frac{{PMMS}_{c,s,r}}{{PMM}_{c,r}}\right]}^{{-\sigma M}_{c,r}}, \forall c\in CE, r\in R, s\in R$$

9 $${PMM}_{c,r}={\left[\sum_{s}{SMS}_{c,s,r}*{{PMMS}_{c,s,r}}^{1{-\sigma M}_{c,r}}\right]}^{\frac{1}{1{-\sigma M}_{c,r}}}, \forall c\in CE, r\in R$$

where $PMMS$ is the market price of import good $c$ into region $r$ from source $s$, $PMM$ is the composite market price of import good $c$ in $r$, $QM$ is the demand for the composite import good $c$ in $r$, and $SMS$ is the value-share of good $c$’s import into $r$ by source $s$. ${\sigma M}_{c,r}$ is the elasticity of substitution of imported good $c$ in $r$ by source.

After sourcing imports, then agents decide on the optimal mix of imported and domestic products (Eq. 10 and 11) based on their relative price levels (Eq. 12).

10 $${QM}_{c,r}={QQ}_{c,r}*{SMQ}_{c,r}*{\left[{PMM}_{c,r}/{PQ}_{c,r}\right]}^{{-\sigma Q}_{c,r}}, \forall c\in CE, r\in R$$

11 $${QD}_{c,r}={QQ}_{c,r}*{SDQ}_{c,r}*{\left[{PY}_{c,r}/{PQ}_{c,r}\right]}^{{-\sigma Q}_{c,r}} , \forall c\in CE, r\in R$$

12 $$\begin{aligned}&{PQ}_{c,r}\\&={\left[\begin{array}{c}{SMQ}_{c,r}*{{PMM}_{c,r}}^{1{-\sigma Q}_{c,r}}\\+{SDQ}_{c,r}*{{PY}_{c,r}}^{1{-\sigma Q}_{c,r}}\end{array}\right]}^{\frac{1}{1{-\sigma Q}_{c,r}}},\\& \forall c\in CE, r \in R\end{aligned}$$

where $PQ$ is the market price of composite good $c$ in region $r$, $QQ$ is the output of composite good $c$ in $r$, and $SMQ$ and SDQ are the value-shares of the import composite and domestic good $c$ in $r$. ${\sigma Q}_{c,r}$ is the elasticity of substitution between domestic and imported good $c$ in $r$.

Final demand for milled rice $c\in CFC$^11^ in region $r$, is the product of population and per-capita demand ${D}_{c,r}$, which is specified as a double log function of income and prices (Eq. 13). ${Z}_{r}$ represents income by region, ${\varphi }_{r}$ is the income demand elasticity, and ${\omega }_{c,g,r}$ is the matrix of own and cross-price demand elasticities.

13 $$\mathrm{log}{D}_{c,r}={\varphi }_{r}\mathrm{*log}{Z}_{r}+{\sum }_{g \in FC}{\omega }_{c,g,r}*\mathrm{log}{PQ}_{g,r} , \forall c\in CFC, r\in R$$

The supply of exogenous intermediate inputs (seeds, fertilizers, pesticides, energy, and water), capital, and labor are specified as perfectly elastic, thus their prices ($PFC$) are treated as constant, exogenous variables. Land is considered the only factor with limited supply. Hence, sectoral output $Y$ is constrained only by the supply of land ${L}_{c,r}$ used in the production of paddy rice, which is represented by a double log function of land rental rates ${PL}_{c,r}$.

14 $$\mathrm{log}{L}_{c,r}={\theta }_{c,r}\mathrm{log}{PL}_{c,r} , \forall c\in CP, r\in R$$

The land own-price supply elasticity ${\theta }_{c,r}$ are calibrated following Keller (1976) to reflect rice supply elasticities found in the literature.

## Footnotes

1 Data from 2008–2010 were missing, providing 15 years of complete data instead of 18.

2 The average yield of IR8 for the pooled sample was not statistically different (*P* > 0.10) than the average yield of all observations of IR8 in the Philippines.

3 While IRRI elite lines are planted globally, obtaining accurate adoption data by year is challenging. As such, the results of this study underestimate the global impacts of the IRRI breeding program.

4 These average replacement rates are consistent with previous studies, which found that the average replacement rate for modern rice varieties in the Philippines was 8–11 years (Laborte et al., 2015). The variety Ciherang (released in 2000 and sown in 17.21% of the total Indonesian area in 2021) has been shown to still be popular among Indonesian rice producers because of its resistance to disease and pests, early maturity, and high productivity despite being 24 years old (Qadir et al., 2024).
